# Metabolite profiling of wheat (*Triticum aestivum* L.) phloem exudate

**DOI:** 10.1186/1746-4811-10-27

**Published:** 2014-08-15

**Authors:** Lachlan James Palmer, Daniel Anthony Dias, Berin Boughton, Ute Roessner, Robin David Graham, James Constantine Roy Stangoulis

**Affiliations:** 1 School of Biological Science, Flinders University, Bedford Park, South Australia 5042, Australia; 2 Metabolomics Australia, School of Botany, The University of Melbourne, Parkville, Melbourne 3010, Victoria, Australia

**Keywords:** Aphid stylectomy, Exudate, Grain loading, GC-MS, LC-MS, Metabolomics, Method development, Phloem, Wheat

## Abstract

**Background:**

Biofortification of staple crops with essential micronutrients relies on the efficient, long distance transport of nutrients to the developing seed. The main route of this transport in common wheat (*Triticum aestivum*) is via the phloem, but due to the reactive nature of some essential micronutrients (specifically Fe and Zn), they need to form ligands with metabolites for transport within the phloem. Current methods available in collecting phloem exudate allows for small volumes (μL or nL) to be collected which limits the breadth of metabolite analysis. We present a technical advance in the measurement of 79 metabolites in as little as 19.5 nL of phloem exudate. This was achieved by using mass spectrometry based, metabolomic techniques.

**Results:**

Using gas chromatography–mass spectrometry (GC-MS), 79 metabolites were detected in wheat phloem. Of these, 53 were identified with respect to their chemistry and 26 were classified as unknowns. Using the ratio of ion area for each metabolite to the total ion area for all metabolites, 39 showed significant changes in metabolite profile with a change in wheat reproductive maturity, from 8–12 to 17–21 days after anthesis. Of these, 21 were shown to increase and 18 decreased as the plant matured. An amine group derivitisation method coupled with liquid chromatography MS (LC-MS) based metabolomics was able to quantify 26 metabolites and semi-quantitative data was available for a further 3 metabolites.

**Conclusions:**

This study demonstrates that it is possible to determine metabolite profiles from extremely small volumes of phloem exudate and that this method can be used to determine variability within the metabolite profile of phloem that has occurred with changes in maturity. This is also believed to be the first report of the presence of the important metal complexing metabolite, nicotianamine in the phloem of wheat.

## Background

Deficiencies of Fe and Zn in humans have been identified as a serious issue of concern for developing countries. In a 2002 World Health Organisation report it was estimated that in 2000, 1.6 million people died as a direct result of Fe and Zn deficiency and a further 60 million healthy life years were lost [1]. Approximately 60% of the health life years lost occurred in developing countries within Africa and South-East Asia [1]. Biofortification of staple crops has been identified as a possible way of combating the issue of micronutrient deficiency [2] and attempts to increase the levels of mineral and vitamin micronutrients in the harvested and edible plant parts using genetic or agronomic techniques is currently underway [3]. An important part of the mineral biofortification process is the transport of these elements from the source to the sink (i.e. from soil, through to the roots, stems and leaves, and then to the seed). Within a plant, the long distance transport pathways of the xylem and phloem are the major routes for nutrient movement to developing seeds [4]. In the case of wheat, the phloem is very important as there is a xylem discontinuity at the base of the grain [5] which results in all macro and micro nutrients first transferring to the phloem before unloading into the grain. During the transport of Fe and Zn in the phloem these minerals must be complexed due to their reactive nature [6]. A variety of metabolites have been theorised to complex Fe, Zn and other essential minerals within the phloem [7]. Of these, nictoianamine and cystine are proposed to play a major role in the modelled transport of Fe and Zn [7], and in rice, nicotianamine has been found to complex Zn in the phloem [8].

Phloem is a complex matrix which consists of water, sugars, amino acids, organic acids, secondary metabolites, peptides and hormones along with ions and a number of macromolecules, including proteins, small RNAs and mRNAs [9,10]. Recent reviews have highlighted the importance of phloem composition in long distance transport and signalling throughout the plant [9,10] and these reviews have also examined the difficulty and issues related to collection of phloem for analysis. There are three main techniques in which phloem can be collected for direct analysis: 1) cutting the stem and collecting the liquid that exudes; 2) making use of an Ethylenediaminetetraacetic acid (EDTA) solution to allow a freshly cut plant part to continue to exude; 3) using insect stylectomy to collect phloem exudate (see [11] for further details). The first two methods have limitations when applied to cereal crops. Cutting the stem for collecting phloem is limited to a small selection of plant species such as castor bean [12] and cucurbits [13] and is not possible for cereals. In wheat, phloem will not exude from cuts made to the stem or leaves under field or glasshouse conditions, however phloem will exude from the grain pedicel after the removal of the seed [14]. This limits the accessibility to wheat phloem and also involves interference with the developing ear. EDTA facilitated exudation also has its limitations, owing to the difficulty in quantifying phloem volume for accurate concentration measurements and also because EDTA facilitated exudation may be contaminated by components from damaged cells other than the phloem and the apoplastic space [11]. Insect stylectomy using aphids and planthoppers has been used to access the phloem of cereal crops for the analysis of some metabolites within the phloem [8,15]. The main limitation of stylectomy based collection is the small volumes involved. With exudation rates ranging from 4.2 to 354 nl h^-1^[16] volumes collected are in the low μl to nl range [16,17]. Due to these small volumes, accurate measurement of phloem collections has been difficult which has limited the scope of metabolomic profiling of the phloem. In most reports of metabolites in phloem collected by stylectomy, collections were made over several hours to enable sufficient volumes to be collected for analysis, as measured using 0.5 μl micro capillaries [8,18]. In more recent work, an alternative technique for measuring phloem volume has been used to measure diurnal variability in amino acid concentrations in volumes as little as 2.1 nl [15]. In this current research, we demonstrate the use of accurate volume measurements for the quantitative analysis of amine-containing metabolites detected by LC-MS and semi-quantitative analysis of the metabolite profile of wheat phloem using GC-MS. We also present the results of semi-quantitative analysis of changes in the metabolite profile during the grain loading period.

## Results

### GC-MS metabolite profiling

GC-MS metabolomic profiling was tested as this has been used previously to profile the metabolites in plant tissues. For example, tissue level changes as a tolerance response to Fe deficiency in peas [19]. Additional file 1: Table S1 details all 79 metabolites identified by GC-MS and for some metabolites, multiple derivatives are created and these are shown in Additional file 1: Table S2 as they were included in calculations of the ion ratio. Of the 79 metabolites identified it was found that 40 had non-normal distributions and attempts were made to transform the data prior to statistical analysis. Of the 40 metabolites, 2 were not able to be transformed to produce a normal distribution (Additional file 1: Table S3) and so were not included in statistical testing.

Of the 79 metabolites detected, there were 26 unknown compounds found and these were not identified using either in-house or commercial libraries nor the GOLM Metabolome Database [20], so they are listed with the following notation. UN1_10.61_158 = Unknown 1 with a retention time of 10.61 minutes with a unique ion at 158 *m/z*. The area of a particular fragment ion was selected and was subsequently adjusted for each sample by dividing it by the volume of phloem collected and then the ratio of this area to the total area for all identified ions in the sample was calculated and used for statistical comparison between different stages during grain loading.

The results from independent student *t*-tests on metabolites showing significant changes between peak grain loading (9–11 DAA) and the end of grain loading (18–20 DAA) are shown in Tables 1 and 2. The results listed in Table 1 show the 18 metabolites had a statistically significant decrease in the phloem as grain loading progressed, from 9–11 DAA to 18–20 DAA. Ornithine had the greatest reduction showing a 4.6 fold decrease. 3-amino-piperidin-2-one, UN08 and Glutamine also declined by 3.5-, 3.4- and 3.4-fold respectively (Table 1). There were another 7 metabolites that had more than a two-fold decrease as grain loading progressed (Table 1).

**Table 1:** **The mean and standard error of the difference and Fold change for metabolites, profiled using GC-MS, that significantly decreased (*p *** **<0.05) in the phloem from 9–11 DAA (n = 15) to 18–20 DAA (n = 16)**

**Metabolite**	**Transformation**	**Levene’s test for equality of variances sig.**	***t*-test for equality of means sig. (2-tailed)**	**Mean difference**	**Std. error difference**	**Fold change**
3-amino-piperidin-2-one 2TMS	SQRT	0.227	0.000	-0.34838	0.07402	-3.4
Alanine 2TMS	SQRT	0.000^un^	0.009	-0.23127	0.08092	-1.9
Arginine 3TMS	Ln	0.286	0.000	-0.84617	0.20727	-2.3
Glutamate 3TMS	None	0.003^un^	0.005	-1.11392	0.35445	-2.3
Glutamine 3TMS	CBRT	0.296	0.001	-0.34581	0.09220	-2.6
Histidine 3TMS	Ln	0.994	0.032	-0.62065	0.27622	-1.9
Homoserine 3TMS	None	0.613	0.000	-0.08141	0.01851	-1.9
Lysine 4TMS	None	0.180	0.006	-0.57860	0.19365	-1.4
Ornithine 3TMS	None	0.001^un^	0.007	-0.17965	0.05689	-4.6
Pyroglutamate 2TMS	None	0.444	0.018	-3.21496	1.28551	-1.3
Serine 3TMS	None	0.073	0.000	-4.85198	1.16680	-1.6
Trehalose 8TMS	Ln	0.952	0.000	-1.25509	0.19795	-3.5
UN01_10.61_158	CBRT	0.360	0.000	-0.07041	0.01492	-2.3
UN07_17.62_275	Ln	0.177	0.002	-0.68564	0.20462	-2.0
UN08_17.96_360	CBRT	0.673	0.001	-0.19065	0.05177	-3.4
UN09_18.15_275	CBRT	0.363	0.003	-0.08408	0.02541	-2.0
UN11_19.48_299	CBRT	0.003^un^	0.003	-0.08195	0.02451	-2.0
UN26_14.48_229	Ln	0.034^un^	0.001	-0.61709	0.15206	-1.9

Also shown are the *p* values for Levene’s test of equality of variance, where values are less than 0.05 (equal variances not assumed = ^un^), equal sample variances were not assumed when calculating *t*-test. (*x*TMS = Trimethylsilyl derivative where *x* = the number of TMS groups; *y*MX = methoxyamine derivatised product where *y* = 1 or 2).

**Table 2:** **The mean and standard error of the difference and Fold change for metabolites, profiled using GC-MS, that significantly increased (*p *** **<0.05) in the phloem from 9–11 DAA (n = 15) to 18–20 DAA (n = 16)**

**Metabolite**	**Transformation**	**Levene’s test for equality of variances sig.**	***t*-test for equality of means sig. (2-tailed)**	**Mean difference**	**Std. error difference**	**Fold change**
Citric acid 4TMS	None	0.512	0.000	0.23250	0.05283	1.8
Fructose_MX1	None	0.002^un^	0.000	0.27792	0.05953	1.8
Fumarate 2TMS	None	0.000^un^	0.004	0.00942	0.00289	1.6
Gluconic acid-1,5-lactone 4TMS	None	0.066	0.002	0.34643	0.09990	1.7
Glucose MX1	None	0.008^un^	0.002	0.63124	0.17488	1.7
Glycine 3TMS	None	0.026^un^	0.000	0.17284	0.03384	2.2
Hexadecanoate 1TMS	SQRT	0.004^un^	0.017	0.27188	0.10404	1.9
Methionine 1TMS	None	0.337	0.024	0.00554	0.00233	1.3
Octadecanoate 1TMS	SQRT	0.028^un^	0.021	0.21070	0.08527	1.8
Phenylalanine 2TMS	None	0.654	0.018	1.07470	0.42754	1.5
Putrescine 4TMS	None	0.697	0.000	0.82294	0.13397	1.9
Quinic acid 5TMS	SQRT	0.010^un^	0.007	0.32413	0.10951	2.5
Shikimic acid 4TMS	SQRT	0.060	0.000	0.23331	0.05785	2.9
Succinate 2TMS	None	0.000^un^	0.010	0.05240	0.01802	2.3
Tyrosine 3TMS	None	0.180	0.000	1.09938	0.24564	1.8
UN04_15.56_185	None	0.000^un^	0.019	0.10668	0.04183	1.8
UN10_19.08_217	None	0.106	0.007	0.44254	0.15290	1.4
UN16_25.71_339	None	0.016^un^	0.015	0.00806	0.00302	1.7
UN17_27.24_375	None	0.003^un^	0.000	0.01570	0.00379	1.8
UN18_28.91_437	None	0.011^un^	0.001	0.01317	0.00358	1.6
UN20_32.34_503	InvCBRT	0.020^un^	0.049	-0.62079	0.29828	1.8

Also shown are the *p* values for Levene’s test of equality of variance, where values are less than 0.05 (equal variances not assumed = ^un^), equal sample variances were not assumed when calculating *t*-test. (*x*TMS = Trimethylsilyl derivative where *x* = the number of TMS groups; *y*MX = methoxyamine derivatised product where *y* = 1 or 2).

There were 21 metabolites that had a significant increase in the phloem as grain loading progressed from 8–12 DAA to 17–21 DAA (Table 2). Of these, shikimic acid had the greatest increase (2.9 fold), while quinic acid, succinate and glycine also had more than a 2 fold increase (2.5, 2.3 and 2.2 respectively, Table 2).

### LC-MS

Quantification of amine group containing metabolites in the phloem exudates was conducted according to Boughten et al. [21]. The concentrations of the metabolites identified are detailed in Table 3. Two metabolites were not able to be quantified due to issues with standard stability affecting the calibration curve and the response relative to the ISTD is presented instead. To the authors knowledge this is the first time that nicotianamine (NA) has been directly quantified. For metabolites where an authentic standard was not available or could not be generated, the ratio of ion area to internal standard was used to generate a relative response. For the LC-MS analysis, phloem collection volumes used were between 43.4 nl and 180.34 nl, with an mean of 95.7 nl ± 61.43. Due to the exploratory nature of this analysis, only four samples were analysed, two from each maturity, and no significant changes were observed (data not shown). Therefore only the average for all samples is presented in Table 3.

**Table 3:** **Amine group containing metabolites identified in the phloem of wheat as measured by LC-MS collected at two maturities (*n *** **=4), unless otherwise stated mean and standard error of all quantitated metabolites are reported as mMol L**^**-1**^

**Metabolite**	**Mean**	**SE**
Ammonia (ISTD RR)	33.8	1.275
Glutathione (ISTD RR)	0.58	0.0905
Glutamine	170.8	50.895
Valine	75.5	17.52
Histidine	64.7	25.24
Serine	63.1	25.455
Glutamate	36.3	15.32
Arginine	39.8	3.74
Alanine	42.0	6.045
Proline	21.2	8.125
Phenylalanine	58.1	18.365
Threonine	49.7	15.515
Isoleucine	44.3	11.275
Leucine	43.7	16.34
Tryptophan	44.1	8.405
Lysine	48.0	10.315
Glycine	39.9	18.275
Tyrosine	27.7	9.495
Aspartic acid	18.7	7.205
Methionine	18.8	8.31
Asparagine	7.4	2.77
β-Alanine	1.7	0.595
GABA	1.1	0.45
Ornithine	1.2	0.58
Citrulline (μmol L^-1^)	232.3	39.14
Nicotianamine (μmol L^-1^)*	255.4	96.71
Cysteine (μmol L^-1^)	70.9	13.91
4-Hydroxy-proline (μmol L^-1^)	38.5	9.675

Metabolites that were measured but did not have an external standard are reported as a ratio of response in relation to the internal standard (ISTD RR). *n = 3, outlier was removed.

## Discussion

The analysis of the metabolite profile of phloem has been restricted in the past due to limitations in the amount collected and the availability and sensitivity of analytical techniques. With exudate flow rates in wheat ranging from 0.07 to 5.9 nl min^-1^[16], volumes that are collected per sample are normally less than a μl. For previous work examining metabolite profiles in the phloem, volume measurement has been done in a variety of ways [22-24]. Phloem volumes or sample amounts are estimated using the length of the liquid within the microcap [24], the weight of sample collected [23] or by collecting to the volume of the microcap [22]. For a series of papers examining metabolites in phloem [25-27], phloem volumes of between 10 and 60 nl were collected using 0.5 μl microcaps but to our knowledge there is no mention of how volume was estimated in the microcap. Even in the original method, these papers all refer to where volumes of 5 nl were collected using a 0.5 μl microcap [28] and there are no details of how the sample volume is derived. The measurement of sample volume is a key part of improving the accuracy of analysis and enables better detection of changes in metabolite profile.

One of the main issues for the accurate measurement of sub-μl volumes is evaporation and work carried out previously has demonstrated an increase in the osmotic potential when phloem samples are collected in air [29,30]. To counter the effects of evaporation, collection of exudate under oil is the accepted method [11] but in more recent work by our laboratory, we have demonstrated that accurate measurement under oil has technical difficulties due to the potential for measurement errors arising from the optical nature of the measurement and the surface shape of the oil used in the collection step [16]. Recent work has made advances in volume measurement: work examining the diurnal effect on the concentration of amino acids in the phloem, made use of a correction factor for air based droplet volume measurements [15]. This technique used measurements made under oil, which reduces the effect of evaporation, as a comparison for collections measured in air and enable a correction factor to be derived. We have further refined this technique of volume measurement using digital photography and software to further reduce the effect of evaporation and to quantify the accuracy of the volume measurement [16]. This method has been used successfully to quantify inorganic components in the phloem of wheat and to detect significant changes with maturity [31]. Oil has also been identified as a potential contaminant for certain metabolite analyses [15], and we have also found that for GC-MS profiling, paraffin oil is a significant contaminant suppressing metabolite signal from the phloem causing an increase in the baseline due to the elution of multiple hydrocarbons present in paraffin oil (refer to Additional file 1 for trace images). This observation was consistent with the GC-MS analysis of petroleum based oil as a contaminant [32].

When using phloem samples that were measured in air we were able to identify, using GC-MS, 79 different metabolites (Additional file 1: Table S1) and of these, 38 showed significant variability in phloem composition with a change in maturity (Tables 1 and 2). Of these metabolites with significant changes, 21 increased and 18 decreased in the phloem as the plant aged. For samples analysed using an LC-MS method specific for amine containing compounds, we were able to identify 30 metabolites and of these quantify the concentration of 27 metabolites within the phloem (Table 3). For the GC-MS metabolite profiling analysis, full quantification of all metabolites is not feasible. This is due to the large number of metabolites detected by GC-MS, making it unfeasible to establish calibration curves for all metabolites detected and so peak area is used for semi-quantitative analysis. Due to the use of peak area, metabolites cannot be compared to one another as the peak area is dependent on the derivitisation process, and all metabolites have a different response factor. For specific metabolites, it may be possible to set up calibration curves for quantification of phloem concentrations by GC-MS and so allow further exploration of results of interest identified from metabolite profiling.

One result of interest is the significant changes in sugars other than sucrose within the phloem. For most work on the phloem, only sucrose is reported [22,33,34]. It has been established that sucrose is the dominant sugar involved in sugar transport [35] and it is assumed that hexoses (glucose and fructose) are not normally present, and when they are, they are mostly seen when using EDTA facilitated exudation [35]. Glucose and fructose have been shown to exist (5.5% and 1.5%) in the phloem of perennial ryegrass (*Lolium perenne* L.) collected from aphid stylectomy [36]. A change in sugar composition was found when plants were defoliated with a decrease of more than 80% for sucrose concentration and decreases of 42% and 47% in glucose and fructose concentrations, respectively [36]. This may indicate a source other than leaf for hexoses within the phloem. The results from the GC-MS analysis highlighted a significant increase in glucose and fructose levels within the phloem during the grain loading period (Table 2).

Of particular interest are the results presented from the LC-MS analysis quantifying NA and demonstrating the presence of glutathione. These two metabolites have been found to play important roles in complexing essential micronutrients during long distance transport in the phloem [7,8,37,38]. In a theoretical model of metabolite and micronutrient speciation in the phloem it was found that 54.4% of Zn was likely to be bound to NA and the remaining Zn complexed with amino acid complexes of cysteine and cysteine with histidine (41.2% and 2.8% respectively) [7]. In the case of Fe, 99% of the ferrous ions would be complexed by NA and only 19.3% of ferric ions would be complexed by NA while the remaining ferric ions would be complexed with glutamate and citrate (70% and 9.2% respectively) [7]. A potential fault with this model is that it does not incorporate 2’-deoxymugineic acid (DMA) as a potential candidate for complex formation. In work performed on the phloem of rice, the concentration of DMA was found to be between 152 μMol L^-1^[39] and 150 μMol L^-1^[8]. This was much higher than the NA values reported of 66 μMol L^-1^[39] and 76 μMol L^-1^[8]. In rice it has been found that the main Zn complex ligand was NA whilst for Fe, DMA was the main metabolite responsible for complexing this metal [8]. Glutathione has been found to play a role in Cd transportation as part of the detoxification process [40]. Glutathione was tested as a chelating agent in the speciation model but was found to complex less than 2% of Zn though it was mentioned that if Cd was included this may affect the model dynamics [7]. In this work we have reported for the first time the concentration of NA in the phloem of wheat with an average concentration of 255.4 μMol L^-1^ ± 96.71 which is within the range reported in castor bean [37,38,41] but is much higher than what has been reported in rice [8,39]. Previous work in our lab has shown a significant increase in Zn and Mg in the phloem during grain loading [31] and it’s also likely that there is an associated increase in metal complexing metabolites. This relationship has been demonstrated in castor bean where the NA phloem concentrations reported 4 and 8 days after imbibition were 206 μMol L^-1^, [38] which is close to what was observed in this work (255.4 μMol L^-1^ ± 96.71). Further exploration of NA flux at different maturities may give further insight into the role of NA in essential micronutrient transport.

It is anticipated that further method development would enable the analysis and quantification of DMA and glutathione within the phloem which would assist in the development of a wheat specific speciation model for the long distance transport of essential micronutrients. The amine binding LC-MS method used here gave in-conclusive results for DMA (data not shown) and a different method is required.

The ability to obtain a profile of a broad range of metabolites is a powerful tool not only for understanding the transport of essential micronutrients to the grain and other vegetative tissues, but also for examining responses to toxic or deficient conditions. An example of this is where it was possible to identify a metabolite complex responsible for boron mobilisation in the phloem [42] that could lead to efficiency in boron utilization [43]. Metabolite profiling has also been used widely on plant tissues to examine tissue level dynamics such as changes involved in tolerance to nutrient deficient conditions such as Fe deficiency in pea [19], and also metabolite variation during the ripening process in capsicum [44]. The methods outlined in this study add further capability to researchers interested in metabolite changes both on a whole plant level during maturation and also when plants are under abiotic or nutrient based stresses.

## Conclusion

This study demonstrates the production of a complex metabolite profile from extremely small volumes of phloem exudate using GC-MS. We also demonstrate that this method of metabolite profiling can be used to determine significant maturity based variability within the metabolite profile of the phloem. To our knowledge this is the first report of the presence of and quantification of NA in the phloem of wheat.

## Materials and methods

### Plant material

Wheat (*Triticum aestivum* L. genotype ‘Samnyt 16’) seedlings were grown in 70x100 mm pots in Debco™ Green Wizard potting mix within a growth room. Growth room conditions were 13/11 h light/dark at 20°C/10°C with a minimum of 400 μmol m^-2^ s^-1^ light at the leaf surface. Plants were transferred to a greenhouse where aphids were applied and kept there for a maximum of 48 h.

### Aphid stylectomy

Aphid stylectomy procedures were adapted from the method established by Downing and Unwin [45]. A short video of the method is presented in Additional file 2 and a summary of the method is as follows. Aphids were taken from an anholocyclic *Sitobion miscanthi* (Indian grain aphid) culture maintained at Flinders University on wheat plants kept under greenhouse conditions. Only apterous aphids were used in the experiments.

Aphids were secured to wheat plants (immediately below the head on the peduncle), a minimum of 12 h prior to stylectomy, using specially prepared cages (refer to Additional file 3 for construction specifications). Plants were watered to saturation at time of aphid caging. Stylectomy was performed using high-frequency micro-cauterisation under a Leica microscope (M165 C or MZ16) using an electrolytically-sharpened tungsten needle in combination with a micromanipulator. Exudate samples were collected using glass micro-capillaries (30–0017, Harvard Apparatus) pulled using a capillary puller (Narishige). The relative humidity during collections ranged from 41% to 50%.

### Microscope measurement of nanolitre phloem exudate volumes

Exudate volumes were measured using the method published in [16]. In brief, exudate flow rates were estimated from photo sequences taken, in air, using a Leica microscope (M165C or MZ16) with an attached camera (DFC295 or DFC280) and the multi-time module from the Leica Application Suite software (v3.6.0). Photo sequences were taken immediately after obtaining an exuding stylet, approximately every 15 minutes during collection and immediately prior to the end of the collection. Photo sequences consisted of five photos with a one second interval between photos. The droplet radius for each photo in a sequence was measured using the interactive measurement module within the Leica Application Suite and an estimate of droplet volume calculated. Using the time interval between each two photos in a sequence the exudation flow rate was estimated from the change in volume between photos. The average of the estimated flow rate from all sequences in each collection was multiplied by the respective collection length to give an estimate of the collection volume. A correction factor, as determined previously, was applied to correct for evaporation [16]. Samples were deposited into 200 μl glass vial inserts (Agilent) containing 5 μl of Millipore Milli-Q™ water (>18.2 MΩ cm^-1^), centrifuged for 20 seconds at 14,000 rpm in a 1.5 ml microcentrifuge tube and insert was transferred to 2 ml auto sampler vials (Agilent) and stored at -80°C. After samples had been collected samples were lyophilized in a freeze dryer prior to shipment for metabolomics profiling and quantitation at Metabolomics Australia, School of Botany, The University of Melbourne. Table 4 shows the average and standard error for the time that sample collection was started, average volume and average maturity (DAA) for the samples allocated to the maturity groupings analysed using LC-MS and GC-MS.

**Table 4:** N, Mean and SE, for both maturity groups (DAA group), for sample collection start times, phloem exudate volumes and number of days after anthesis for samples analysed by GC-MS and LC-MS

**Analysis**	**Variable**	**DAA Group**	**N**	**Mean**	**SE**
GC-MS	collection start	8-12 DAA	16	16:02:33.75	0:06:50.17
		17-21 DAA	20	15:35:45.00	0:11:25.50
	Corrected vol	8-12 DAA	16	90.6	13.26
		17-21 DAA	20	62.3	6.91
	DAA	8-12 DAA	16	9.9	0.22
		17-21 DAA	20	18.5	0.17
LC-MS	collection start	8-12 DAA	2	15:04:30	0:08:30
		17-21 DAA	2	14:58:00	1:24:00
	Corrected vol	8-12 DAA	2	140.6	39.79
		17-21 DAA	2	50.9	7.48
	DAA	8-12 DAA	2	10.0	0.00
		17-21 DAA	2	18.5	0.50

### GC-MS analysis of phloem exudate

GC-MS analysis of phloem exudate samples was carried out using a method modified from [46]. The lyophilized phloem samples were re-dissolved in 5 μL of 30 mg mL^-1^ methoxyamine hydrochloride in pyridine and derivatised at 37°C for 120 min with mixing at 500 rpm. The samples were then treated for 30 min with 10 μL *N*,*O*-*bis*-(trimethylsilyl)trifluoroacetamide (BSTFA) and 2.0 μL retention time standard mixture [0.029% (*v*/*v*) *n* dodecane, *n*-pentadecane, *n*-nonadecane, *n*-docosane, *n*-octacosane, *n*-dotriacontane, *n*-hexatriacontane dissolved in pyridine] with mixing at 500 rpm. Each derivatised sample was allowed to rest for 60 min prior to injection.

Samples (1 μL) were injected into a GC-MS system comprised of a Gerstel 2.5.2 autosampler, a 7890A Agilent gas chromatograph and a 5975C Agilent quadrupole MS (Agilent, Santa Clara, USA). The MS was adjusted 171 according to the manufacturer’s recommendations using *tris*-(perfluorobutyl)-amine (CF43). The GC was performed on a 30 m VF-5MS column with 0.2 μm film thickness and a 10 m Integra guard column (Agilent J&W GC Column). The injection temperature was set at 250°C, the MS transfer line at 280°C, the ion source adjusted to 250°C and the quadrupole at 150°C. Helium was used as the carrier gas at a flow rate of 1.0 mL min^-1^. For the polar metabolite analysis, the following temperature program was used; start at injection 70°C, a hold for 1 min, followed by a 7°C min^-1^ oven temperature, ramp to 325°C and a final 6 min heating at 325°C. Both chromatograms and mass spectra were evaluated using the Chemstation Data Analysis program (Agilent, Santa Clara, USA). Mass spectra of eluting compounds were identified and validated using the public domain mass spectra library of Max-Planck-Institute for Plant Physiology, Golm, Germany (http://csbdb.mpimp-golm.mpg.de/csbdb/dbma/msri.html) and the *in-house* Metabolomics Australia mass spectral library. All matching mass spectra were additionally verified by determination of the retention time by analysis of authentic standard substances. Resulting relative response ratios, that is, selected ion area of each metabolite was normalized to phloem volume for each identified metabolite. For metabolites which had multiple TMS derivatives, normalized ion areas were presented.

### LC-MS analysis of phloem exudate

Quantification of amine containing metabolites in nl phloem exudate was modified from the method reported in Boughton et al. [21]. To the lyophilized phloem sample 4 μl of Borate buffer (200 mM at pH 8.8 with 1 mM ascorbic acid, 10 mM tris(2-carboxyethyl)phosphine (TCEP) and 25 μM 2-aminobutyric acid (added as an ISTD) was added, then 1 μl of 10 mM 6-Aminoquinolyl-N-hydroxysuccinimidylcarbamate (AQC, dissolved in 100% acetonitrile). The glass inserts were sealed with parafilm then left to rest at room temperature for 30 minutes. Analysis and quantification by LC-MS was done as previously reported [21].

### Statistical analysis

Statistics including Student’s *t*-test were calculated using IBM SPSS statistics software (version 22). A normal distribution was determined to achieved when the z-scores for the skew and kurtosis were less than 1.96 which the cut off indicating a significant skew or kurtosis at the 95% level [47]. Of the metabolites identified, 40 were found to have significant skew or kurtosis to their distribution. These metabolites were transformed using the following transformations in increasing power; 16 using square root (SQRT), 8 using cube root (CBRT), 11 using the natural logarithm (Ln) and 3 using the inverse cube root (InvCBRT). A further 2 metabolites were unable to be transformed successfully. For the Student’s *t*-test as calculated using SPSS, results are produced with and without the assumption of equal variance between treatments and the Levene’s statistic gives an indication if this assumption is met or rejected.

## Abbreviations

GC-MS: Gas chromatography - mass spectrometry; LC-MS: Liquid chromatography - mass spectrometry; EDTA: Ethylenediaminetetraacetic acid; DAA: days after anthesis; ISTD: Internal standard.

## Competing interests

The authors declare that they have no competing interests.

## Authors’ contributions

LJP helped in conceiving study, collected phloem samples, did ICP-MS analysis, statistical analysis and drafted the manuscript. DAD assisted with GC-MS method development, acquisition and data analysis of phloem samples. BB assisted with LC-MS method development, running of sample and data analysis. UR helped in the design of the study, and GC-MS and LC-MS method development. RG helped in the design of the study and drafting of manuscript. JS participated in conception and design of the study and assisted with drafting of manuscript. All authors read and approved the final manuscript.

## Supplementary Material

Additional file 1 Further information on GC-MS analysis, including example of separation traces with and without oil contamination, full list of metabolites detected, replicated derivatives and un-normalisable metabolites. Click here for file

Additional file 2 Short video outlining the method of aphid stylectomy. Click here for file

Additional file 3 Specifications and method for constructing aphid cages. Click here for file
