# Supplementary material for: Metabolite profiling of wheat (Triticum aestivum L.) phloem exudate
Source: Plant Methods. 2014 Aug 15;10:27. doi: 10.1186/1746-4811-10-27 (PMC4138413; doi:10.1186/1746-4811-10-27)
Supplement: Additional file 1 — Further information on GC-MS analysis, including example of separation traces with and without oil contamination, full list of metabolites detected, replicated derivatives and un-normalisable metabolites. [file 1746-4811-10-27-S1.docx]

Supplementary Table 1: Mean and standard area for metabolites identified using GC-MS that were normally distributed or could be transformed to a normal distribution (CBRT = cube root, SQRT = square root, Ln = natural logarithm and InvCBRT = inverse cube root). . (xTMS = Trimethylsilyl derivative where x = the number of TMS groups; yMX = methoxyamine derivatised product where y = 1 or 2)

| Metabolite | Transformation | DAA group | N | Mean | Std. Error |
| --- | --- | --- | --- | --- | --- |
| 3-amino-piperidin-2-one 2TMS | SQRT | 8-12 DAA | 15 | .7601 | .06047 |
| 3-amino-piperidin-2-one 2TMS | SQRT | 17-21 DAA | 16 | .4117 | .04389 |
| 3-hydroxybenzoic acid 2TMS | CBRT | 8-12 DAA | 13 | .2105 | .01345 |
| 3-hydroxybenzoic acid 2TMS | CBRT | 17-21 DAA | 14 | .2514 | .01692 |
| 4-aminobutyric acid 3TMS | SQRT | 8-12 DAA | 15 | .7184 | .10467 |
| 4-aminobutyric acid 3TMS | SQRT | 17-21 DAA | 16 | 1.0876 | .21333 |
| 4-hydroxybenzoic acid 2TMS | None | 8-12 DAA | 15 | .2074 | .02783 |
| 4-hydroxybenzoic acid 2TMS | None | 17-21 DAA | 16 | .2746 | .03079 |
| Alanine 2TMS | SQRT | 8-12 DAA | 15 | .8469 | .03328 |
| Alanine 2TMS | SQRT | 17-21 DAA | 16 | .6156 | .07376 |
| Arginine 3TMS | Ln | 8-12 DAA | 15 | -2.3801 | .16441 |
| Arginine 3TMS | Ln | 17-21 DAA | 16 | -3.2263 | .12859 |
| Asparagine_3TMS | Ln | 8-12 DAA | 15 | -1.4471 | .32846 |
| Asparagine_3TMS | Ln | 17-21 DAA | 16 | -2.3324 | .33139 |
| Aspartate 3TMS | None | 8-12 DAA | 6 | 5.3105 | 1.38125 |
| Aspartate 3TMS | None | 17-21 DAA | 3 | 3.6118 | 1.02767 |
| Beta-alanine 3TMS | SQRT | 8-12 DAA | 12 | .1174 | .01309 |
| Beta-alanine 3TMS | SQRT | 17-21 DAA | 15 | .1617 | .02326 |
| Caffeic acid 3TMS | None | 8-12 DAA | 0 |  |  |
| Caffeic acid 3TMS | None | 17-21 DAA | 1 | .0114 |  |
| Citric acid 4TMS | None | 8-12 DAA | 15 | .2936 | .04216 |
| Citric acid 4TMS | None | 17-21 DAA | 16 | .5261 | .03249 |
| Fructose_MX1 | None | 8-12 DAA | 15 | .3369 | .02531 |
| Fructose_MX1 | None | 17-21 DAA | 16 | .6148 | .05388 |
| Fructose-1-phosphate | None | 8-12 DAA | 6 | .0117 | .00305 |
| Fructose-1-phosphate | None | 17-21 DAA | 6 | .0129 | .00291 |
| Fructose-6-phosphate MX1 | CBRT | 8-12 DAA | 15 | .4880 | .01737 |
| Fructose-6-phosphate MX1 | CBRT | 17-21 DAA | 16 | .4932 | .01761 |
| Fumarate 2TMS | None | 8-12 DAA | 14 | .0155 | .00103 |
| Fumarate 2TMS | None | 17-21 DAA | 15 | .0250 | .00270 |
| Gluconic acid-1,5-lactone 4TMS | None | 8-12 DAA | 15 | .4741 | .05412 |
| Gluconic acid-1,5-lactone 4TMS | None | 17-21 DAA | 16 | .8205 | .08230 |
| Gluconic acid-6-phosphate 7TMS | None | 8-12 DAA | 14 | .0286 | .00311 |
| Gluconic acid-6-phosphate 7TMS | None | 17-21 DAA | 15 | .0317 | .00370 |
| Glucose MX1 | None | 8-12 DAA | 15 | .9486 | .07653 |
| Glucose MX1 | None | 17-21 DAA | 16 | 1.5798 | .15725 |
| Glucose-6-phosphate MX1 | Ln | 8-12 DAA | 15 | -2.2746 | .17445 |
| Glucose-6-phosphate MX1 | Ln | 17-21 DAA | 16 | -2.1634 | .16818 |
| Glutamate 3TMS | None | 8-12 DAA | 15 | 2.0037 | .30789 |
| Glutamate 3TMS | None | 17-21 DAA | 16 | .8898 | .17562 |
| Glutamine 3TMS | CBRT | 8-12 DAA | 15 | 1.2640 | .05148 |
| Glutamine 3TMS | CBRT | 17-21 DAA | 16 | .9182 | .07506 |
| Glyceric-3-phosphate 4TMS | SQRT | 8-12 DAA | 15 | .3931 | .02371 |
| Glyceric-3-phosphate 4TMS | SQRT | 17-21 DAA | 16 | .3794 | .02192 |
| Glycerol-3-phosphate 4TMS | Ln | 8-12 DAA | 13 | -2.7953 | .09540 |
| Glycerol-3-phosphate 4TMS | Ln | 17-21 DAA | 6 | -3.8822 | .57176 |
| Glycine 3TMS | None | 8-12 DAA | 14 | .1390 | .01467 |
| Glycine 3TMS | None | 17-21 DAA | 16 | .3118 | .03049 |
| Hexadecanoate 1TMS | SQRT | 8-12 DAA | 15 | .7464 | .03921 |
| Hexadecanoate 1TMS | SQRT | 17-21 DAA | 16 | 1.0182 | .09637 |
| Histidine 3TMS | Ln | 8-12 DAA | 15 | -.4729 | .19310 |
| Histidine 3TMS | Ln | 17-21 DAA | 16 | -1.0936 | .19685 |
| Homoserine 3TMS | None | 8-12 DAA | 15 | .1692 | .01478 |
| Homoserine 3TMS | None | 17-21 DAA | 16 | .0878 | .01138 |
| Isoleucine 2TMS | None | 8-12 DAA | 15 | 4.3845 | .33189 |
| Isoleucine 2TMS | None | 17-21 DAA | 16 | 4.6155 | .47652 |
| Itaconic acid 2TMS | None | 8-12 DAA | 6 | .0014 | .00030 |
| Itaconic acid 2TMS | None | 17-21 DAA | 3 | .0016 | .00064 |
| Lysine 4TMS | None | 8-12 DAA | 15 | 1.9889 | .15594 |
| Lysine 4TMS | None | 17-21 DAA | 16 | 1.4103 | .11748 |
| Malic acid 3TMS | SQRT | 8-12 DAA | 15 | .4628 | .04707 |
| Malic acid 3TMS | SQRT | 17-21 DAA | 16 | .5671 | .07449 |
| Methionine 1TMS | None | 8-12 DAA | 15 | .0192 | .00141 |
| Methionine 1TMS | None | 17-21 DAA | 16 | .0248 | .00183 |
| Myoinositol 6TMS | None | 8-12 DAA | 15 | .2662 | .05483 |
| Myoinositol 6TMS | None | 17-21 DAA | 16 | .2513 | .05204 |
| Octadecanoate 1TMS | SQRT | 8-12 DAA | 15 | .6156 | .03947 |
| Octadecanoate 1TMS | SQRT | 17-21 DAA | 16 | .8263 | .07558 |
| Ornithine 3TMS | None | 8-12 DAA | 15 | .2302 | .05609 |
| Ornithine 3TMS | None | 17-21 DAA | 16 | .0505 | .00953 |
| Orotic acid 3TMS | InvCBRT | 8-12 DAA | 10 | 9.3858 | 1.89067 |
| Orotic acid 3TMS | InvCBRT | 17-21 DAA | 14 | 7.1621 | 1.50739 |
| Phenylalanine 2TMS | None | 8-12 DAA | 15 | 2.3270 | .31111 |
| Phenylalanine 2TMS | None | 17-21 DAA | 16 | 3.4017 | .29378 |
| Pipecolic acid 2TMS | Ln | 8-12 DAA | 15 | -3.2029 | .17966 |
| Pipecolic acid 2TMS | Ln | 17-21 DAA | 16 | -3.8537 | .31272 |
| Proline 2TMS | Ln | 8-12 DAA | 15 | .6249 | .37268 |
| Proline 2TMS | Ln | 17-21 DAA | 16 | -.2399 | .30230 |
| Putrescine 4TMS | None | 8-12 DAA | 15 | .9030 | .09908 |
| Putrescine 4TMS | None | 17-21 DAA | 16 | 1.7259 | .09056 |
| Pyroglutamate 2TMS | None | 8-12 DAA | 15 | 13.7620 | .87668 |
| Pyroglutamate 2TMS | None | 17-21 DAA | 16 | 10.5470 | .93457 |
| Quinic acid 5TMS | SQRT | 8-12 DAA | 15 | .5504 | .05124 |
| Quinic acid 5TMS | SQRT | 17-21 DAA | 16 | .8746 | .09678 |
| Serine 3TMS | None | 8-12 DAA | 15 | 12.8278 | .62200 |
| Serine 3TMS | None | 17-21 DAA | 16 | 7.9758 | .96699 |
| Shikimic acid 4TMS | SQRT | 8-12 DAA | 15 | .3357 | .03053 |
| Shikimic acid 4TMS | SQRT | 17-21 DAA | 16 | .5690 | .04811 |
| Succinate 2TMS | None | 8-12 DAA | 15 | .0412 | .00517 |
| Succinate 2TMS | None | 17-21 DAA | 16 | .0936 | .01726 |
| Sucrose 8TMS | SQRT | 8-12 DAA | 15 | .8533 | .06927 |
| Sucrose 8TMS | SQRT | 17-21 DAA | 16 | 1.0708 | .11593 |
| Threonine 3TMS | None | 8-12 DAA | 15 | 2.2151 | .11628 |
| Threonine 3TMS | None | 17-21 DAA | 16 | 2.0685 | .17744 |
| Trehalose 8TMS | Ln | 8-12 DAA | 15 | .1658 | .13531 |
| Trehalose 8TMS | Ln | 17-21 DAA | 16 | -1.0892 | .14365 |
| Tryptophan 2TMS | SQRT | 8-12 DAA | 15 | .4206 | .03662 |
| Tryptophan 2TMS | SQRT | 17-21 DAA | 16 | .4632 | .04185 |
| Tyrosine 3TMS | None | 8-12 DAA | 15 | 1.3150 | .13691 |
| Tyrosine 3TMS | None | 17-21 DAA | 16 | 2.4144 | .20012 |
| UN01_10.61_158 | CBRT | 8-12 DAA | 15 | .2915 | .00931 |
| UN01_10.61_158 | CBRT | 17-21 DAA | 16 | .2211 | .01151 |
| UN02_14.04_350 | SQRT | 8-12 DAA | 15 | .1929 | .00727 |
| UN02_14.04_350 | SQRT | 17-21 DAA | 16 | .2054 | .00651 |
| UN03_14.36_320 | Ln | 8-12 DAA | 15 | -4.0163 | .18504 |
| UN03_14.36_320 | Ln | 17-21 DAA | 16 | -3.6552 | .12732 |
| UN04_15.56_185 | None | 8-12 DAA | 15 | .1343 | .01763 |
| UN04_15.56_185 | None | 17-21 DAA | 16 | .2410 | .03793 |
| UN06_17.16_259 | None | 8-12 DAA | 15 | .0020 | .00020 |
| UN06_17.16_259 | None | 17-21 DAA | 11 | .0020 | .00027 |
| UN07_17.62_275 | Ln | 8-12 DAA | 15 | -2.4859 | .10686 |
| UN07_17.62_275 | Ln | 17-21 DAA | 16 | -3.1716 | .17081 |
| UN08_17.96_360 | CBRT | 8-12 DAA | 15 | .5695 | .03943 |
| UN08_17.96_360 | CBRT | 17-21 DAA | 16 | .3788 | .03387 |
| UN09_18.15_275 | CBRT | 8-12 DAA | 15 | .4077 | .01499 |
| UN09_18.15_275 | CBRT | 17-21 DAA | 16 | .3237 | .02019 |
| UN10_19.08_217 | None | 8-12 DAA | 15 | 1.1104 | .07687 |
| UN10_19.08_217 | None | 17-21 DAA | 16 | 1.5529 | .12923 |
| UN11_19.48_299 | CBRT | 8-12 DAA | 15 | .3965 | .00888 |
| UN11_19.48_299 | CBRT | 17-21 DAA | 16 | .3146 | .02284 |
| UN13_25.04_130 | None | 8-12 DAA | 3 | .2134 | .06297 |
| UN13_25.04_130 | None | 17-21 DAA | 13 | .5824 | .11004 |
| UN14_25.08_503 | None | 8-12 DAA | 15 | .2324 | .03511 |
| UN14_25.08_503 | None | 17-21 DAA | 16 | .1901 | .02124 |
| UN15_25.55_425 | None | 8-12 DAA | 15 | .0136 | .00090 |
| UN15_25.55_425 | None | 17-21 DAA | 16 | .0164 | .00113 |
| UN16_25.71_339 | None | 8-12 DAA | 15 | .0115 | .00111 |
| UN16_25.71_339 | None | 17-21 DAA | 16 | .0195 | .00281 |
| UN17_27.24_375 | None | 8-12 DAA | 15 | .0200 | .00170 |
| UN17_27.24_375 | None | 17-21 DAA | 16 | .0357 | .00338 |
| UN18_28.91_437 | None | 8-12 DAA | 15 | .0204 | .00145 |
| UN18_28.91_437 | None | 17-21 DAA | 16 | .0336 | .00327 |
| UN19_29.64_437 | None | 8-12 DAA | 15 | .0450 | .00856 |
| UN19_29.64_437 | None | 17-21 DAA | 16 | .0665 | .00798 |
| UN20_32.34_503 | InvCBRT | 8-12 DAA | 15 | 3.6351 | .25733 |
| UN20_32.34_503 | InvCBRT | 17-21 DAA | 16 | 3.0143 | .15083 |
| UN21_32.89_387 | None | 8-12 DAA | 15 | .0140 | .00180 |
| UN21_32.89_387 | None | 17-21 DAA | 16 | .0132 | .00079 |
| UN22_33.13_513 | SQRT | 8-12 DAA | 15 | .1429 | .01043 |
| UN22_33.13_513 | SQRT | 17-21 DAA | 16 | .1421 | .00409 |
| UN23_33.43_517 | None | 8-12 DAA | 15 | .0186 | .00204 |
| UN23_33.43_517 | None | 17-21 DAA | 16 | .0184 | .00102 |
| UN24_33.79_423 | None | 8-12 DAA | 15 | .0604 | .00655 |
| UN24_33.79_423 | None | 17-21 DAA | 16 | .0735 | .00438 |
| UN25_33.99_373 | SQRT | 8-12 DAA | 15 | .1250 | .00865 |
| UN25_33.99_373 | SQRT | 17-21 DAA | 16 | .1301 | .00390 |
| UN26_14.48_229 | Ln | 8-12 DAA | 15 | -.8407 | .06452 |
| UN26_14.48_229 | Ln | 17-21 DAA | 16 | -1.4578 | .13769 |
| Urea 2TMS | CBRT | 8-12 DAA | 15 | .5322 | .01650 |
| Urea 2TMS | CBRT | 17-21 DAA | 16 | .5813 | .02667 |
| Valine 2TMS | None | 8-12 DAA | 15 | 7.2086 | .49262 |
| Valine 2TMS | None | 17-21 DAA | 16 | 6.9817 | .59083 |
| Xylofuranose 4TMS | None | 8-12 DAA | 15 | .7413 | .12641 |
| Xylofuranose 4TMS | None | 17-21 DAA | 16 | 1.1156 | .15085 |
|  |  |  |  |  |  |
|  |  |  |  |  |  |

Supplementary Table 2: replicated derivatives also identified by GC-MS

| Metabolite | Transformation | DAA group | N | Mean | Std. Error |
| --- | --- | --- | --- | --- | --- |
| Fructose_MX2 | None | 8-12 DAA | 15 | .3369 | .02531 |
| Fructose_MX2 | None | 17-21 DAA | 16 | .6148 | .05388 |
| Glucose MX2 | None | 8-12 DAA | 15 | .1978 | .01321 |
| Glucose MX2 | None | 17-21 DAA | 16 | .3147 | .02588 |
| Glutamine 4TMS | InvCBRT | 8-12 DAA | 15 | 2.8886 | .24264 |
| Glutamine 4TMS | InvCBRT | 17-21 DAA | 15 | 3.6201 | .20492 |
| Glycine 2TMS | None | 8-12 DAA | 15 | .0254 | .00363 |
| Glycine 2TMS | None | 17-21 DAA | 16 | .0254 | .00214 |
| INSD Docosane | None | 8-12 DAA | 15 | 1.1212 | .13186 |
| INSD Docosane | None | 17-21 DAA | 16 | 1.8162 | .23873 |
| Lysine 3TMSa | None | 8-12 DAA | 15 | .3489 | .04061 |
| Lysine 3TMSa | None | 17-21 DAA | 16 | .1438 | .02457 |
| Lysine 3TMSb | None | 8-12 DAA | 15 | .0994 | .01055 |
| Lysine 3TMSb | None | 17-21 DAA | 16 | .0870 | .00457 |
| Methionine 2TMS | None | 8-12 DAA | 14 | .6500 | .14253 |
| Methionine 2TMS | None | 17-21 DAA | 16 | .7569 | .08826 |
| Serine 2TMS | None | 8-12 DAA | 15 | .0284 | .00362 |
| Serine 2TMS | None | 17-21 DAA | 16 | .0240 | .00266 |
| Threonine 2TMS | None | 8-12 DAA | 15 | .1472 | .01394 |
| Threonine 2TMS | None | 17-21 DAA | 16 | .1308 | .01853 |
| Tryptophan 3TMS | SQRT | 8-12 DAA | 15 | .4440 | .04685 |
| Tryptophan 3TMS | SQRT | 17-21 DAA | 16 | .5530 | .06559 |

Supplementary Table 3: Metabolites that couldn’t be transformed to produce a normal distribution.

|  | | | | |
| --- | --- | --- | --- | --- |
| metabolite | DAA group | N | Mean | SE |
| UN12_20.05_370 | 8-12 DAA | 15 | .0039 | .00051 |
| UN12_20.05_370 | 17-21 DAA | 16 | .0071 | .00040 |
| UN5_16.19_299 | 8-12 DAA | 14 | .0992 | .01637 |
| UN5_16.19_299 | 17-21 DAA | 15 | .0603 | .01172 |


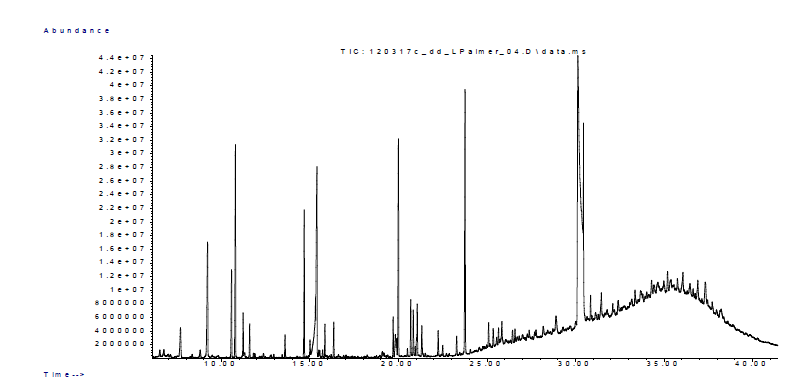


Figure 1: Phloem sample measured in oil. Note the baseline deviation starting at approximately 23 minutes.

Figure 2: phloem sample measured in air. Note the comparatively flat baseline with no deviation in the second half of the run.
