# Supplementary material for: Metabolite profiling of wheat (Triticum aestivum L.) phloem exudate
Source: Plant Methods. 2014 Aug 15;10:27. doi: 10.1186/1746-4811-10-27 (PMC4138413; doi:10.1186/1746-4811-10-27)
Supplement: Additional file 3 — Specifications and method for constructing aphid cages. [file 1746-4811-10-27-S3.docx]

**Supplementary material 2: Aphid Cages**


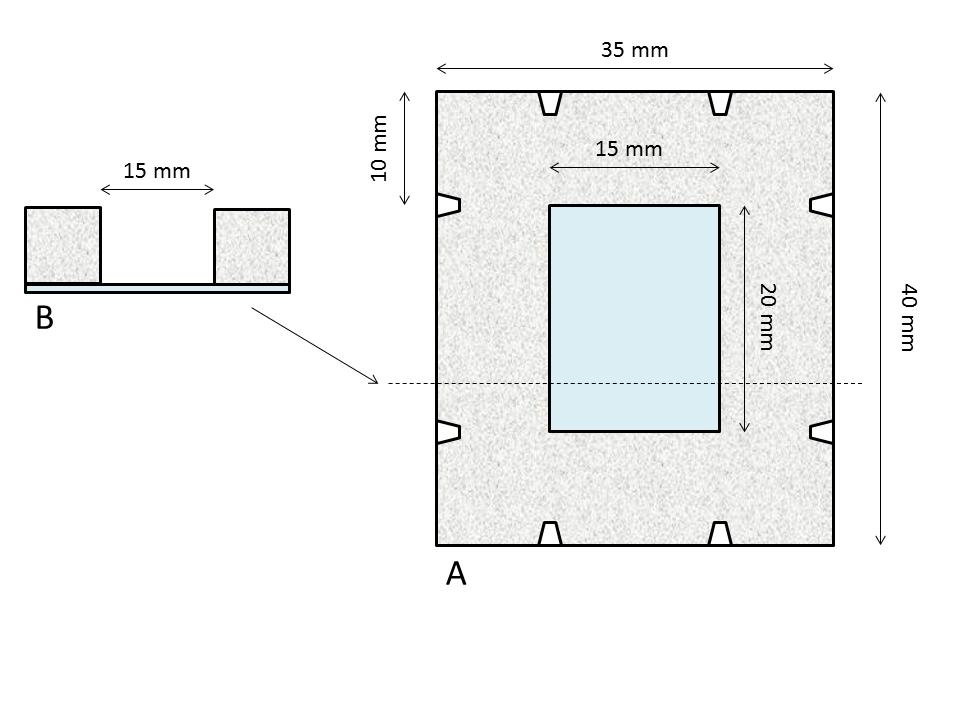


Figure 1: specifications for construction of aphid cages. (A) is top down view of one half of cage, (B) is view of cross section at dotted line on (A).

Materials:

- Polyethylene (PE) “peeled” foam, 12 mm thick (Clark Rubber, Australia)
- Stiff plastic sheet, Rigid PVC sheet 0.5 mm thick (Plastic Centre, Australia) or 0.8 mm thick polypropylene sheet (Eckersley’s art and craft, Australia) or PVC plastic from waste packaging (for example container of Agilent boxed vials (5182-0714)).
- Plastics glue (Selleys, Australia)

Cage Construction:

1. Cut plastic sheet to size as specified in Fig 1
2. Cut foam to same size as in 1. and cut hole in foam as specified in Fig 1 (blue shaded section of A).
3. Glue foam and plastic together (Plastic surface needs to be primed with primer pen, prior to glue application)
4. When dry, cut notches 10 mm in from each corner
5. Repeat steps 1 to 4 for other half of cage.
